# Correction: JARID1B promotes metastasis and epithelial-mesenchymal transition via PTEN/AKT signaling in hepatocellular carcinoma cells

**DOI:** 10.18632/oncotarget.27470

**Published:** 2020-05-12

**Authors:** Bo Tang, Guangying Qi, Fang Tang, Shengguang Yuan, Zhenran Wang, Xingsi Liang, Bo Li, Shuiping Yu, Jie Liu, Qi Huang, Yangchao Wei, Run Zhai, Biao Lei, Hongping Yu, Xingyuan Jiao, Songqing He

**Affiliations:** ^1^ Department of Hepatobiliary Surgery, Guilin Medical University, Affiliated Hospital, Guilin, Guangxi, People’s Republic of China; ^2^ Laboratory of Liver Injury and Repair Molecular Medicine, Guilin Medical University, Guilin, Guangxi, People’s Republic of China; ^3^ Department of Pathology and Physiopathology, Guilin Medical University, Guilin, Guangxi, People’s Republic of China; ^4^ Department of Epidemiology and Statistics, School of Public Health, Guilin Medical College, Guilin, Guangxi, People’s Republic of China; ^5^ Department of General Surgery, The First Affiliated Hospital, Sun Yat-Sen University, Guangzhou, People’s Republic of China

**This article has been corrected:** In Figure 5C, “pBabe’s Migration” was accidentally duplicated as “pcDNA3.1’s Migration” in Figure 8D. The corrected Figure 8D is shown below. The authors declare that these corrections do not change the results or conclusions of this paper.

Original article: Oncotarget. 2015; 6:12723–12739. 12723-12739. https://doi.org/10.18632/oncotarget.3713

**Figure 8:**
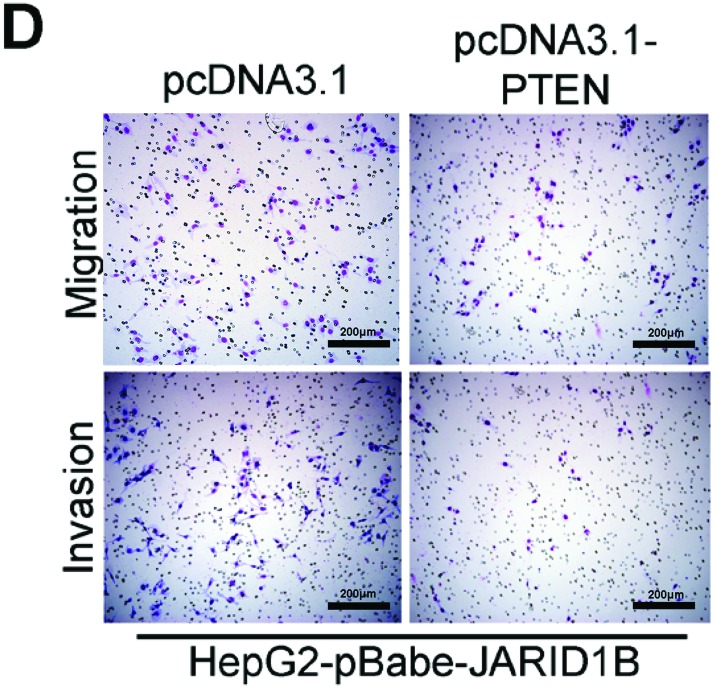
JARID1B regulates PTEN transcriptional expression through H3K4 trimethylation.

(**A** and **B**), the abundance of H3 lysine methylation was assessed in HCC cells with JARID1B overexpression (A) or silencing (B) by Western blotting using whole-cell lysate; total H3 and β-actin were used as a loading control. (**C**) schematic presentation of three regions relative to the PTEN transcriptional start site used as primers to test histone occupied abundance. (**D** and **E**) qChIP was performed to assess H3K4me3 occupancy in HepG2-pBabe-JARID1B (D), SK-Hep1-pSuper-shJARID1B (E) or their control cells. IgG was used as negative control (D and E, left). “Percentage of input” indicates the ratio of DNA fragment of each promoter region bound by H3K4me3 to the total amount of input DNA fragment without H3K4me3 antibody pull-down. **, *P* < 0.01 is based on the Student *t* test. All results are from three independent experiments. Error bars, SD.

